# Supplementary material for: Electronic Structure and Lithium Diffusion in LiAl2(OH)6Cl Studied by First Principle Calculations
Source: Molecules. 2019 Jul 23;24(14):2667. doi: 10.3390/molecules24142667 (PMC6681411; doi:10.3390/molecules24142667)
Supplement: Supplementary file 1 [file molecules-24-02667-s001.pdf]

Electronic Structure and Lithium Diffusion in $LiAl_2(OH)_6Cl$ Studied by First Principle Calculations

Yueping Zhang[1], Xiyue Cheng[1], Chen Wu[1], Jürgen Köhler[1, 2], Shuiquan Deng[1, *]

[1] State Key Laboratory of Structural Chemistry, Fujian Institute of Research on the Structure of Matter (FJIRSM) Chinese Academy of Sciences (CAS) Fuzhou, 350002, China

[2] Max-Planck-Institute for Solid State Research, Heisenbergstr. 1, D-70569 Stuttgart, Germany

* Correspondence: sdeng@fjirsm.ac. Tel.:+86-0591-6317-3252

Table S1 Crystal data of $LiAl_2(OH)_6Cl$

**Item**		**Experiment [1]**	**After relaxation**
**Space group**		$P6_3/mcm$ (193)	-
**Lattice**		a = b = 5.10 Å, c =14.2994 Å; α = β = 90°; γ = 120°	A = b = 5.158 Å, c = 14.2588 Å; α=β=90°; γ=120°
**Wyckoff Position,**	**Li**	2b, (0, 0, 0)	2b, (0, 0, 0)
**Atomic position**	**Al**	4d, (1/3, 2/3, 0)	4d, (1/3, 2/3, 0)
	**Cl**	2a, (0, 0, 1/4)	2a, (0, 0, 1/4)
	**O**	12k, (0.6350, 0.6350, 0.5672)	12k, (0.6325, 0.6325, 0.5685)
	**H**	12k, (0.6879, 0.6879, 0.6335)	12k, (0.6879, 0.6879, 0.6335)

Table S2 Calculated geometrical parameters for $LiAl_2(OH)_6Cl$.

**Item**	**Experimental value (Å)**	**Calculated value (Å)**	**Item**	**Experimental value (°)**	**Calculated value (°)**
$d_{Li\text{-}O}$	2.095	2.132	∠O-Li-O	79.4~100.6	79.3~100.7
$d_{Al\text{-}O}$	1.888	1.907	∠O-Al-O	77.5~96.4	77.3~96.1
$d_{H...Cl}$	2.304	2.300	∠O-H…Cl	152.2	153.8
$d_{O...Cl}$	3.209	3.208	∠H-O-Li	101.4	99.50

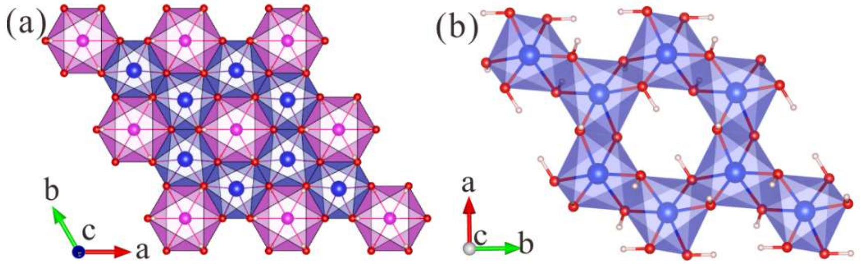

Figure S1 Projection of a) the (2×2×1) layer of the structure of $LiAl_2(OH)_6Cl$ along the [001] direction; b) layer (1×2×1) of the structure showing only the $Al_2(OH)_6$ part

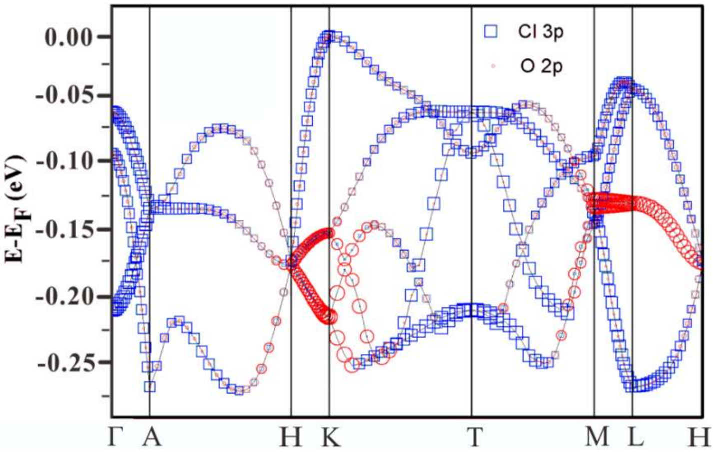

Figure S2 Orbital-resolved band structure of $LiAl_2(OH)_6Cl$ from -0.3 to 0 eV

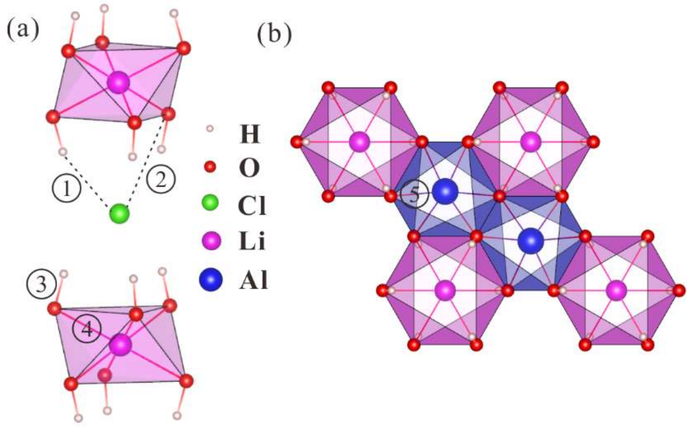

Figure S3 The arrangement of atom pairs in the structure of of $LiAl_2(OH)_6Cl$ corresponding to the COHP plots (see Fig. 2c) with ① for Cl-H, ② for Cl-O, ③ for H-O ④ for Li-O and ⑤ for Al-O, respectively.

Reference

1. Besserguenev, A.V.; Fogg, A.M.; Francis, R.J.; Price, S.J.; O'Hare, D.; Isupov, V.P.; Tolochko, B.P. *Chem. Mater.* **1997**, *9*, 241-247.
